# 

**Published:** 1958-04

**Authors:** 


MEDICAL JOURNAL OF THE SOUTH-WEST

*(Incorporating the Bristol Medico-Chirurgical Journal)*

Established 1883

VOL. 73 (ii) April 1958 NO. 268

### *Contents*

| | | Page |
|---|---|---|
| DISEASES IN SEARCH OF VIRUSES . | *Prof. C. H. Stuart-Harris* | 29 |
| PAIN RELATED TO THE TEETH AND JAWS . | *Prof. A. I. Darling* | 36 |
| SUPPORTIVE TREATMENT OF ACUTE COR PULMONALE DUE TO MASSIVE PULMONARY EMBOLISM | *Dr. E. A. W. Houghton* | 41 |
| ACCIDENTAL POISONING WITH THE ANTIHISTAMINE DRUG "HISTANTIN" . . . . | *Clinical Pathological Conference* | 45 |
| APOLOGY . . . . . . . . . . | | 49 |
| CORRESPONDENCE . . . . . . . | | 50 |
| NOTES AND NEWS . . . . . . . | | 51 |
| APPOINTMENTS . . . . . . . . | | 53 |

*PUBLISHED QUARTERLY*

*ANNUAL SUBSCRIPTION 16/- POST FREE*

PUBLISHED UNDER THE AUSPICES OF THE
BRISTOL MEDICO-CHIRURGICAL SOCIETY

### Editorial Committee

Dr. J. E. CATES, Department of Medicine, Bristol Royal Infirmary. *Editor.*
Dr. N. J. BROWN, Southmead Hospital, Bristol. *Assistant Editor.*
Dr. J. MACRAE, Ham Green Hospital.
Dr. R. C. WOFINDEN, Central Clinic, Bristol.
Mr. A. E. S. ROBERTS, Medical Library, University of Bristol. *Secretary.*

### *Local Correspondents*

*Bath* . . Mr. A. D. BATEMAN, 3 The Circus, Bath.
*Exeter* . . Dr. L. N. JACKSON, Mount Jocelyn, Crediton, Devon.
*Gloucester* . Dr. R. F. JARRETT, 9 College Green, Gloucester.
*Plymouth* . Dr. C. R. CROFT, Lexden, Hartley Av., Plymouth.
*Taunton* . Dr. M. MCALLEY, 1 The Crescent, Taunton.
*Torquay* . Dr. A. ROBINSON THOMAS, 20 Devon Square, Newton Abbot, Devon.
*Truro* . . Dr. F. D. M. HOCKING, St. Andrews, Perranporth, Cornwall.
*Yeovil* . . Mr. J. A. VERE NICOLL, Knapp House, Yeovil, Somerset.

### NOTICE TO CONTRIBUTORS

Original articles are invited, provided they have not been published elsewhere. Notes of forthcoming events, meetings held, degrees conferred, staff appointments, and other local news are welcomed. The editorial committee reserves the right to make literary corrections.

Illustrations should always be supplied ready for reproduction. All re-touchings or other operations required will be charged to the author. Line drawings should be drawn in Indian ink. We regret that we must ask authors to pay for making blocks, if this is necessary.

J. W. ARROWSMITH LTD., WINTERSTOKE ROAD,
BRISTOL.

### *Notes and News*

Mr. R. V. Cooke has been elected President of the Section of Surgery of the Royal Society of Medicine.

Dr. G. Walters has been appointed whole-time consultant pathologist to the Wolverhampton group of hospitals.

*M.R.C.P.*: P. C. Farrant, M.B. (Brist.).

*Crosby Leonard Price:* R. C. Hodge.

*Mackie Pathology Prize:* R. T. Marcus and J. M. Slater (jointly).

*Aberdeen Graduates Reunion.* Will Aberdeen graduates in this region please send their names and addresses to Professor G. Lennon, University of Bristol.

### NOTICE

LECTURE-DEMONSTRATIONS FOR GENERAL PRACTITIONERS

*Sunday Mornings at* 10.30 *a.m.*

13th Apr.: Department of Medicine, Bristol Royal Infirmary, **"Virus Diseases"**, J. MaCrae, M.D., CH.B., F.R.E.P.S., D.P.H.

20th. Apr.: Department of Medicine, Bristol Royal Infirmary, **"Certification of Unfitness for work"**, I. E. Phelps, M.R.C.S., L.R.C.P., D.C.H.

27th Apr.,: Department of Medicine, Bristol Royal Infirmary, **"Backache"**, K. H. Pridie, M.B., C.H.B., B.S., F.R.C.S.

4th May: Department of Medicine, Bristol Royal Infirmary, **"Iatrogenic Diseases"**, Professor C. Bruce Perry.

11th May: Department of Medicine, Bristol Royal Infirmary, **"Selection of Ulcer patients for Surgery"**, T. H. Butler, M.B., CH.B., F.R.C.S. and J. M. Naish, M.A., M.D., B.CHIR., F.R.C.P.

18th May: Bristol Eye Hospital, **"Demonstration of Normal and Abnormal Fundi"**, Anthony Palin, M.A., M.B., B.CHIR, F.R.C.S.E.

25th May: Whit Sunday—No Lecture.

1st June: Barrow Hospital, Barrow Gurney, **"Addicts and Psychopaths"**, R. E. Hemphill, M.A., M.D., B.A.O., D.P.M.

8th June: Department of Medicine, Bristol Royal Infirmary, **"Epilepsies"**, A. M. G. Campbell, D.M., F.R.C.P.

15th June: Bristol Maternity Hospital, **"Maternal Mortality, part ii"**, H. L. Shepherd, M.B., CH.M., F.R.C.O.G.

*Applications to attend should be made to the Medical Postgraduate Dean, University of Bristol.*

### *Appointments*

### *UNITED BRISTOL HOSPITALS*

*December 1957—February 1958*

| *Name* | *Appointment* | *Formerly* |
|---|---|---|
| NICOL, W. A., L.D.S., R.F.P.S. (GLASGOW), D.D.O., (GLASGOW), DIPLOMA IN ORTHODONTICS (R.C.S.). | Consultant Orthodontist. | Assistant Orthodontist, Bristol Dental Hospital. |
| PHILLIPS, C. I., F.R.C.S., M.D. (ABERDEEN), D.O., R.C.S., P., ENG. | Consultant Opthalmic Surgeon. | Senior Registrar, St. Thomas's Hospital. |
| HORTON, R. E., M.B.E., M.S. (LONDON), F.R.C.S. (ENG.) | Consultant Surgeon. | Lecturer in Surgery, Bristol University. Hon. Assistant Surgeon, United Bristol Hospitals. |
| PANIKKAR, MISS T., M.B., B.S., D.A. | Registrar in Anaesthetics. | S.H.O. in Anaesthetics, Hitchin, Herts. |

### *BRISTOL*

| *Name* | *Appointment* | *Formerly* |
|---|---|---|
| SEAR, A. J., L.D.S., B.D.S. (B'HAM.) | Registrar to the Maxillo-Facial Unit at Frenchay Hospital and in the University of Bristol Dental Hospital. | Dental Officer, Public Health Department, City of Norwich. |
| BANNERMAN, H., M.B., B.S. (DURHAM), D.O.M.S. | Ophthalmologist, Bristol Clinical Area (School Clinics). | Recently (1957), Temporary Senior Registrar, Oxford Eye Hospital. |
| COULSTING, H.S., M.B., CH.B. (MANCHESTER), D.P.M. (MANCHESTER). | Consultant Psychiatrist (Child Guidance), Bristol Clinical Area. | Medical Director of Child Guidance Services to Cheltenham, Gloucester and County Child Guidance Service. |
| SEAGER, C. P., B.SC. (WALES), M.B., B.CH. (WALES), D.P.M. (LONDON). | Assistant Psychiatrist, Bristol Mental Hospitals Group. | Senior Registrar, Bristol Mental Hospitals. |
| COOPER, A. B., M.B., CH.B. (MANCHESTER), D.P.M. (CONJOINT BOARD). | Senior Registrar, Bristol Mental Hospitals. | Psychiatric Registrar, St. John's Hospital, Stone, Nr. Aylesbury, Bucks. |
| NEWSAM, P. N., M.B., B.CH. B.A.O. (BELFAST), D.P.H. D.M.R.D. | Senior Registrar in Radiodiagnosis, Southmead Hospital, Bristol. | Registrar in Radiodiagnosis, Royal Victoria Hospital, Belfast. |

### *NORTH GLOUCESTERSHIRE*

| *Name* | *Appointment* | *Formerly* |
|---|---|---|
| SHILLITO, P. W., M.R.C.S., L.R.C.P. | General Practioner Anaesthetist, Stroud General Hospital (2 sessions). | Is in general practice in Stroud. |
| LEITCH, D. S., M.B., B.S. (SYDNEY). | Surgical Registrar, Cheltenham General Hospital. | S.H.O. Birmingham Accident Hospital. |
| MESSENT, D. O. H., M.B., B.S. (SYDNEY). | Surgical Registrar, Gloucestershire Royal Hospital, Gloucester. | Holding same post on locum tenens basis. |
| LEWIS, J.W., M.B., B.S. (LONDON), D. (OBST), R.C.O.G. | Clinical Assistant in Gynaecology, Stroud General Hospital (1 session). | Is in general practice in Nailsworth. |

### *BATH*

| *Name* | *Appointment* | *Formerly* |
|---|---|---|
| FALLA, H. P. L., M.B., B.S. (LONDON), D.A. (ENGLAND), D. (OBST.) R.C.O.G. | Anaesthetic Registrar, Bath Group of Hospitals. | S.H.O. Anaesthetist, Bath Group of Hospitals. |
| O'FLYNN, B. P., M.B., B.S. B.A.O. (N.U.I.). | Registrar in Pathology, Bath Group of Hospitals. | Pathologist in charge Laboratory at B.M.H., Iserlohn, R.A.M.C. |

### *DEVON AND CORNWALL*

| *Name* | *Appointment* | *Formerly* |
|---|---|---|
| ANKLESARIA, R. P., M.B., B.S. (BOMBAY). | Anaesthetic Registrar, Royal Devon & Exeter Hospital, Exeter. | House Physician, Northampton General Hospital. |
| SIMPSON, J. H., M.A., M.D. (CANTAB), M.R.C.P. (LONDON). | Consultant Physician with an interest in Geriatrics, Exeter Clinical Area. | Assistant Physician in Geriatrics, Norfolk and Norwich Hospital. |
| EDDY, H. D., B.M., B.CH. (OXON). | Clinical Assistant in General Medicine, West Cornwall Clinical Area (5 sessions). | Is in general practice in Truro and also holds appointment as Clinical Assistant in Neurology (2 sessions). |
| LIDDLE, W. W. J., M.B., B.S. (QUEENSLAND), F.R.C.S. (EDINBURGH). | Surgical Registrar, Torbay Hospital, Torquay and Newton Abbot Hospital. | Locum tenens appointment at Edinburgh Royal Infirmary. |
| CHURCHWARD, S. L., M.B., B.S. (SYDNEY). | Surgical Registrar, Royal Cornwall Infirmary, Truro. | Surgical Registrar, Otley General Hospital, Yorks. |
| PHILLIPPS, W. O. S., M.B., CH.B. (OTAGO, N.Z.), F.R.C.S. (EDIN.). | Surgical Registrar, South Devon and East Cornwall Hospital, Plymouth. | Surgical Registrar, Queen Mary's Hospital for Children, Carshalton. |
| PIGGOT, J., M.B., B.CH., B.A.O. (BELFAST), F.R.C.S. (EDIN.). | Research Assistant in the Registrar grade at the Princess Elizabeth Orthopaedic Hospital, Exeter. | Registrar to the Orthopaedic Service, Exeter Clinical Area. |
| RAI, M. Y., M.B., B.S. (MADRAS), F.R.C.S. (EDIN. AND ENG.). | Registrar to the Orthopaedic Service, Exeter Clinical Area. | S.H.O. in Orthopaedics, Royal Cornwall Infirmary, Truro. |

